# What motivates academics toward entrepreneurship? Examining the formation of academic entrepreneurial intention from the push-pull perspective

**DOI:** 10.3389/fpsyg.2023.1073866

**Published:** 2023-02-27

**Authors:** Zhaoyuan Yu, Kangyin Lu

**Affiliations:** Business School, Northeast Normal University, Changchun, Jilin, China

**Keywords:** academic entrepreneurship, entrepreneurial intention, push-pull factor, social network, entrepreneurial motivation

## Abstract

**Introduction:**

Although academic entrepreneurship has received considerable attention over the last decades, little attention has been devoted to discussing the formation process of academic entrepreneurial intention underlying the push-pull perspective. This study attempts to explore the mechanism of how academic entrepreneurial intention is shaped, with job-related negative elements as push factors, and entrepreneurship-related positive attractors as pull factors.

**Methods:**

In this paper, regression analysis and Bootstrap were conducted using SPSS 26.0 and MPLUS 7.0, whose applicability has been widely demonstrated in research.

**Results:**

Findings were derived from 1042 academics from Chinese universities. Results showed that both push and pull factors do contribute to boosting academic entrepreneurial intention. Particularly, the entrepreneurship-related pull factors including entrepreneurial opportunity identification and expected entrepreneurial benefits play a dominant role in trigging academic intention to engaging entrepreneurship. Moreover, our findings further confirmed the moderating role of social network in the process of academics pushed by negative job-related factors to emerge entrepreneurial intention.

**Discussion:**

This study extends the research perspective on the factors influencing academic entrepreneurial intention by examines the impact of push and pull factors on academic entrepreneurial intention simultaneously. This deepens the formation mechanism of academic entrepreneurial intention. Besides, the current study identifies a new look at the role that social network plays in academic entrepreneurship.

## 1. Introduction

Academic entrepreneurship applies the frontier knowledge from universities into entrepreneurial practice, enhancing the translation efficiency of university research outputs, and has been seen as an important “driving force” for industrial upgrading and economic development (Galati et al., 2020; Gieure et al., 2020; Kong et al., 2020). Research on the topic of academic entrepreneurship has received joint attention from the practice and academic communities. Practically, in recent years in China, despite a succession of policies implicated, only a rare percentage of scholars choose to pursue academic entrepreneurship (Wang et al., 2022). This policy–reality discrepancy then raises an important and necessary theoretical question that is urgent to address: exposed to the same stimulus, why do some people develop the intention to engage in entrepreneurship while others do not? The first step in the academic entrepreneurial process is an intention, a sense of readiness to start a business. It is well-proven that entrepreneurial intention is the single best predictor of entrepreneurial behavior (Urban and Chantson, 2019). Only the entrepreneurial intention is triggered, may the act of academic entrepreneurship take place (Ajzen, 1991), which further demonstrates the necessity of investigating the formation mechanism of academic entrepreneurial intention. Existing literature has devoted considerable efforts to understanding the shape of academic entrepreneurial intention (Abreu and Grinevich, 2013; Neves and Brito, 2020; Zhao et al., 2020).

Prior studies category the factors that influence academic entrepreneurial intention into four aspects: demographic characteristics (Urban and Chantson, 2019; Martínez-Martínez et al., 2021), motivations (Holley and Watson, 2017; Johnson et al., 2017), social capital (Wu et al., 2015; Dai and Xu, 2021), and human capital (Lyu et al., 2021). Among these four categories of factors, as motivation and social capital carry the burden of illustrating why and how to start a business, they have been identified as the key factors influencing academic entrepreneurial intention, and have received extensive attention from the current literature (Zhao et al., 2020). In regards to motivation, Aparicio et al. (2016) suggest entrepreneurial opportunity identification is the necessary condition for spawning academic entrepreneurial intention. Foo et al. (2016) believe expected entrepreneurial benefits including raising income, achieving career success, and enhancing social reputation, are fundamental drivers to ignite academic entrepreneurial intention. As such, according to the view of push-pull theory (Suh and Kim, 2018; Haldorai et al., 2019), existing studies so far have mainly explored the positive motivators regarding entrepreneurship on academic entrepreneurial intention from the pull perspective. Nevertheless, individuals are not only affected by the pull but also by the push forces in their decision-making process (Kirchberger and Pohl, 2016; Haldorai et al., 2019).

Academic entrepreneurship, as the transformation process of university teachers from academics toward entrepreneurs, is naturally influenced by push and pull factors. As for the basis of variable selection, entrepreneurial motivation theory points out that the formation of individual entrepreneurial behavior decisions is usually influenced by two kinds of factors: one is the factors related to an individual’s current job, and the other is the factors related to entrepreneurial activities (Obschonka et al., 2015). Combining with the academic entrepreneurship, the former reflects the push effect of job-related negative factors on entrepreneurial intention, and the latter reflects the pull effect of entrepreneurship-related positive factors on entrepreneurial intention (Lee, 2021). Furthermore, existing studies have identified job stress and job dissatisfaction as the dominant job-related negative factors, while entrepreneurial opportunities and expected benefits are the main entrepreneurial-related pull factors that attract academics to engage in academic entrepreneurship (Gambardella et al., 2015). Thus, this paper selects job stress and job dissatisfaction as push factors, entrepreneurial opportunity identification and expected entrepreneurial benefits as pull factors.

Social network, as a major form of social capital, has also received ample attention for its irreplaceable role in the formation process of academic entrepreneurial intention (Fernandez-Perez et al., 2015; Greven et al., 2020; Zhao et al., 2020). For instance, Fritsch and Krabel (2012) propose that resources and information that academics receive from social network could contribute to enhancing academic entrepreneurial intention. Wu et al. (2015) emphasize that there is a tight link between social network and academics’ willingness to start a new business. However, the current research in the field of academic entrepreneurial intention has mainly focused on the direct effect of social network (Zhao et al., 2020). In fact, in the academic’s entrepreneurial decision-making process, social network could act as an essential facilitator or constraint (Diánez-González and Camelo-Ordaz, 2019). Social network can alleviate the information and financial barriers for academics who want to escape from their current job and those who are attracted to entrepreneurial opportunities and rewards. In this case, social network would catalyze the process of push-pull factors affecting academic entrepreneurial intention. Nevertheless, much of the existing work has adopted a narrow focus on the direct predicting effect of social network on academics’ willingness to engage in academic entrepreneurship (Wright and Phan, 2018; Greven et al., 2020). A relative paucity of field research has explored the boundary conditions of the influence of social network on academic entrepreneurial intention from the perspective of entrepreneurial motivation.

Overall, under a dual “push-pull” perspective, this paper explores push and pull factors’ direct and indirect effects on academic entrepreneurial intention, and examines the moderating effect of social network within this process. The empirical analysis is conducted underlying a sample of 1,042 academics from China, using stepwise regression and bootstrap methods. This study contributes to existing research from the following aspects. First, this study extends the research perspective on the factors influencing academic entrepreneurial intention by focusing specifically on the push factors that have been neglected by existing studies. Second, this paper examines the impact of push and pull factors on academic entrepreneurial intention simultaneously, and compares the effects of them, thereby pinpointing the key factors involved. This deepens the formation mechanism of academic entrepreneurial intention. Third, the current study investigates the moderating effect of social network in the formation of academic entrepreneurial intention, which identifies a new look at the role that social network plays in academic entrepreneurship.

## 2. Theory background and hypotheses

The push-pull theory was first proposed by Ravenstein (1885) and initially applied in the field of migration research. The push-pull theory argues that the decision to migrate between two domains is shaped by the push of the original domain and the pull of the entry domain (Haldorai et al., 2019). Nowadays, the push-pull theory has been applied extensively in entrepreneurship research (Harms et al., 2014; Patrick et al., 2016). The push-pull theory also fits in explaining academic entrepreneurship. Because academic entrepreneurship is not a random event or simple transfer of human resources, but a complex and dynamic migration process. In this process, academics’ entrepreneurial intentions would be pushed by job-related negative factors and pulled by entrepreneurship-related positive factors (Agarwal et al., 2016). As such, in this study, we address the formation of academic entrepreneurial intention from the dual push-pull perspective. Specifically, we identified “push factors” as job stress and job dissatisfaction, “pull factors” as entrepreneurial opportunities identification, and expected entrepreneurial benefits.

### 2.1. Job stress, job dissatisfaction, and academic entrepreneurial intention — From the push perspective

Distinct from employee entrepreneurship in enterprises, academics have their own technical achievements, and academic entrepreneurship is a process in which scholars rely on their research achievements to establish enterprises and realize value creation (Neves and Brito, 2020). Academic entrepreneurial intention refers to academics’ inclination to start businesses based on their research achievements (Fernandez-Perez et al., 2015). The intention to engage in academic entrepreneurship would be pushed by negative job-related factors, such as job stress and job dissatisfaction (Holley and Watson, 2017).

Job stress is a stressful reaction or psychological state of the academics to external stimuli caused by the content or atmosphere of the current job (Tartari et al., 2014; Chen, 2019). Academics’ job stress may arise from the long-term stimulation of stressors such as peer competitions. Title rating and scientific research funding, which may drive scholars to develop a desire to start their businesses (Zhang et al., 2019; Wu et al., 2022).

First, recent years in China, growing numbers of academics are under high-level workloads. Peer competition for academics is becoming fiercer, and the pressure for them to advance in titles is getting stronger (Wu et al., 2022). The stress of peer competition and title promotion could drive academics to become entrepreneurs, thus generating a willingness for academic entrepreneurship (Johnson et al., 2017). Second, scholars may also face the pressure of research funding in their current jobs. To obtain the required funds, scholars may try to industrialize their research achievements, and thus develop the desire to engage in academic entrepreneurial activities (Holley and Watson, 2017). In addition, job stress may cause negative impacts on the physical and mental well-being of academics (Obschonka et al., 2015). To relieve the negative consequences of job stress, academics may shift their focus and develop academic entrepreneurial intention (Johnson et al., 2017). Therefore, we assume that:

*H1a*: Job stress positively affects academic entrepreneurial intention.

Job dissatisfaction refers to a frustrated or passive emotional state that academics hold regarding their work (Abbas et al., 2014). Academics who are dissatisfied with their jobs may become unenthusiastic about their work, feel confused about their career development, and sprout academic entrepreneurial intention (Skaalvik and Skaalvik, 2017).

On the one hand, job dissatisfaction in academics may derive from a lack of job challenge and a low level of self-actualization. These scholars may choose to engage in academic entrepreneurial activities to pursue a more challenging job and make full use of their research achievements, thus developing academic entrepreneurial intention (Blaese et al., 2021). On the other hand, compared to entrepreneurs, university teachers usually have lower incomes. Academics dissatisfied with their job incomes may be inclined to devote more effort to other activities that seek more rewards, and thus develop a desire for academic entrepreneurship (Walter et al., 2018). Furthermore, when university teachers suffer from a high level of job dissatisfaction, they may be disappointed and no longer passionate about their current work (Skaalvik and Skaalvik, 2017). This segment of academics tends to devote more energy and time to other activities and may develop academic entrepreneurial intention. Thus, we hypothesize that:

*H1b*: Job dissatisfaction positively affects academic entrepreneurial intention.

Job stress not only exerts a direct influence on academic entrepreneurial intention but also may have an indirect effect on academic entrepreneurial intention through the mediating effect of job dissatisfaction.

For one thing, excessive stress may lead to scholar role overload, burnout, and emotional exhaustion, which naturally reduces the academic’s satisfaction with their current job (Obschonka et al., 2015). The dissatisfaction with university jobs would subsequently boost the academic’s willingness to engage in entrepreneurship activities. For the other thing, the pressure of peer competition and title promotion could trigger the generation of negative emotions such as cognitive bias and anxiety about the career, which could in turn discourage scholars from completing their current works, and then a willingness for academic entrepreneurship would be generated (Blaese et al., 2021). Therefore, this study proposes that:

*H1c*: Job dissatisfaction exerts a mediating effect on the relationship between job stress and academic entrepreneurial intention.

### 2.2. Entrepreneurial opportunity identification, expected entrepreneurial benefits, and academic entrepreneurial intention — From the pull perspective

Entrepreneurial opportunity identification is the procedure by which potential entrepreneurs adopt a creative process to generate business ideas and filter out suitable opportunities (Aparicio et al., 2016). The identification of entrepreneurial opportunity is the very beginning of the entrepreneurial process, and it could foster academic entrepreneurial intention in the following two ways (Fernández-Pérez et al., 2014).

First, academic entrepreneurial intention will not be initiated without a cause, it is usually the result of a vague entrepreneurial idea (Vandor and Franke, 2016; Ruiz-Palomino and Martínez-Cañas, 2021). Unlike employee entrepreneurship in enterprises, academic entrepreneurs are nested in the maternal university, where knowledge production and application take place simultaneously. This assists academics in sensitizing themselves to academic entrepreneurial opportunities. In addition, university teachers usually have abundant research achievements, which enable scholars to generate some entrepreneurial ideas (Wood et al., 2017). After the profitability and innovativeness of these entrepreneurial ideas have been judged, scholars may develop a willingness to start a business.

Second, academics usually keep up with the frontiers of industrial development and the most advanced technologies. Besides, many university teachers also have experience in cooperating with enterprises and can understand their technological needs (Clough et al., 2019). This latest industrial and technological knowledge, as well as the understanding of corporate needs, can help university teachers effectively identify entrepreneurial opportunities thus developing the desire to engage in academic entrepreneurial activities (Gambardella et al., 2015). Thus, we assume that:

*H2a*: Entrepreneurial opportunity identification positively affects academic entrepreneurial intention.

Expected entrepreneurial benefits, refer to academics’ judgments about the probability of financial, reputational, and self-fulfillment rewards that may be achieved by engaging in entrepreneurial activities (Fernández-Pérez et al., 2014). The expected entrepreneurial benefits do play a vital role in the formation process of the academics’ willingness to engage in academic entrepreneurship.

First, the expected entrepreneurial financial benefits would affect academics’ intention to take part in entrepreneurial activities. If academics anticipate that there are substantial financial rewards to be earned by entrepreneurship, then they are more likely to hold a strong desire to start their own businesses (Hahn, 2020). Second, the expected reputational benefits also enhance academics’ entrepreneurial inclination. In cases where scholars anticipate that entrepreneurship helps to enhance their social status and raise their social reputation, they may develop a high-level intention to engaging entrepreneurship (Walter et al., 2018). Finally, academics developing entrepreneurial intention may also be influenced by expected entrepreneurial self-fulfillment (Foo et al., 2016). Academics may undertake entrepreneurship for the sake of gaining spiritual satisfaction. Thus, we hypothesize that:

*H2b*: Expected entrepreneurial benefits positively affect academic entrepreneurial intention.

Entrepreneurial opportunity identification not only has a direct effect on academic entrepreneurial intention but also may have an indirect effect on academic entrepreneurial intention through the mediating effect of expected entrepreneurial benefits.

First, the identification of entrepreneurial opportunities is the initial step in the formation of academic entrepreneurial decisions (Wood et al., 2017). The evaluation of entrepreneurial returns needs to be premised on the identification of entrepreneurial opportunities (Walter et al., 2018). Only when a specific opportunity is identified, can academics accurately assess the likelihood and level of its future rewards, they may develop academic entrepreneurial intention.

Second, the process of entrepreneurial opportunity identification is hardly stripped away from the evaluation of the specific opportunity (Clough et al., 2019). A well-developed entrepreneurial opportunity integrates feasibility and potential rewards. When academics identify an entrepreneurial opportunity that is worth undertaking, they would hold an increased belief in its potential for high-level entrepreneurial returns as well, and then generate high levels of entrepreneurial intentions (Vandor and Franke, 2016). Therefore, we propose that:

*H2c*: Entrepreneurial expected benefit exerts a mediating effect in the relationship between entrepreneurial opportunity identification and academic entrepreneurial intention.

### 2.3. The comparison of push-pull factors on academic entrepreneurial intention

This study has posited that academic entrepreneurial intention is influenced by both job-related push factors and entrepreneurship-related pull factors. In this section, we assume that in comparison to push factors, pull factors dominate in the formation process of academic entrepreneurial intention.

First, the identification of entrepreneurial opportunities is a prerequisite for the emergence of academic entrepreneurial intention (Gambardella et al., 2015). Only when academics identify a feasible entrepreneurial opportunity, can a certain level of academic entrepreneurial intention emerge (Aparicio et al., 2016). In the case of academics simply suffer from push factors such as job stress and job dissatisfaction, while do not identify entrepreneurial opportunities, academic entrepreneurial intention is still likely to remain low.

Second, academic entrepreneurial activity contains a certain degree of risk (Urban and Chantson, 2019). When considering whether to engage in academic entrepreneurial activity, academics may be prudent in comparing the potential gains as well as losses that academic entrepreneurial activity may bring to them. Only academics perceive that entrepreneurial activity could bring them significant rewards, are they likely to develop a desire for academic entrepreneurship. If academics are only pushed by job stress and job dissatisfaction, the willingness for academic entrepreneurship may not be high either (Clough et al., 2019). Thus, this paper hypothesizes that:

*H3*: Pull factors exert stronger effects on academic entrepreneurial intention than push factors.

### 2.4. The moderating effect of social network

Social network represents a type of relational network composed of individuals and organizations, which could provide crucial channels for the acquisition of information and resources (Diánez-González and Camelo-Ordaz, 2019). In contrast to employee entrepreneurship in companies, the connections and experience of collaborating with companies accumulated by academics during their work in universities help to build social networks. According to the social network theory, social network can assist in establishing connections with enterprises, helping academics understand the entrepreneurial process, obtain entrepreneurial resources, and build entrepreneurial connections (Neves and Brito, 2020). Zhao et al. (2020) further argue that social network, as a source of entrepreneurial resources and information, may to a larger extent, act as a “catalyst” to academic entrepreneurial intention. Thus, echoing the above view, we suggest that social network as a source of resources, information and funding channels, could have a catalytic effect on the emergence of academic entrepreneurial intention.

First of all, this study considers that social network positively moderates the impact of job stress on academic entrepreneurial intention. On the one hand, according to social network theory, as the source of entrepreneurial related information, resources, and financial support, social network may empower scholars under job stress with the ability to imagine academic entrepreneurial matters (Diánez-González and Camelo-Ordaz, 2019). This would tackle the key challenge issue of entrepreneurship and facilitate the nurturing and stimulation of confidence, which in turn would stimulate the academics to have greater intentions to start new businesses.

On the other hand, social network could also provide valuable entrepreneurial experiences for academics in high-pressure work environments (Fritsch and Krabel, 2012). Social network theory suggests that individuals connected in a network will communicate with each other. Academics might gain an understanding of the entrepreneurial process and obtain guidance from other members of the social network who have academic entrepreneurial experience. It would help academics learn from the entrepreneurial experiences of others and plan their own pathway to academic entrepreneurship, thus developing a higher willingness for academic entrepreneurship (Greven et al., 2020). Therefore, we assume that:

*H4a*: Social network exerts a moderating effect on the relationship between job stress and academic entrepreneurial intention.

Besides, this study also assumes that social network positively moderates the effect of job dissatisfaction on academic entrepreneurial intention. First, in cases where academics are dissatisfied with their current jobs, as social network theory proposes, academics can use the interpersonal relationships built by social network to solve the essential financing challenge in entrepreneurship. Under the guarantee of the social network, resource and financial barriers to entrepreneurial activities would be reduced and the transformation process of academics to entrepreneurs would become smoother.

Second, as professionals in a traditional non-commercial environment, academics are less exposed to information related to the creation and development of businesses (Huyghe et al., 2016). For those academics who are dissatisfied with their current job and have the desire to start their own business, social network can provide necessary entrepreneurial information and valuable entrepreneurial advice to reinforce and strengthen their entrepreneurial ideas. As such, we suppose that:

*H4b*: Social network exerts a moderating effect on the relationship between job dissatisfaction and academic entrepreneurial intention.

Regarding pull factors, this study assumes that social network reinforces the positive effect of entrepreneurial opportunity identification on academic entrepreneurial intention. First, social network could provide a wide range of social connections, social network theory believes that these connections facilitate the acquisition and flow of information and resources. This can help academics exploit entrepreneurial opportunities (Diánez-González and Camelo-Ordaz, 2019). In this way, academics are allowed take shortcuts to identify high-quality and actionable entrepreneurial opportunities. Faced with these attractive entrepreneurial opportunities, academics would have increased enthusiasm and a higher intention to engage in academic entrepreneurial activities.

Second, social network could provide adequate information, resources, and funding channels to scholars who have identified entrepreneurial opportunities. These essential entrepreneurial resources could enhance the scholars’ confidence in academic entrepreneurial activities, thus developing a higher entrepreneurial intention (Dai and Xu, 2021). Thus, we assume that:

*H5a*: Social network exerts a moderating effect on the relationship between entrepreneurial opportunity identification and academic entrepreneurial intention.

In addition, this study assumes that social network positively moderates the effect of expected entrepreneurial benefits on academic entrepreneurial intention. First, academics in the campus atmosphere may be vague about the expected benefits of entrepreneurship. Social network could reach out to people from the business network, who could offer academics fresh and practical advice on the future benefits of starting a business (Zhao et al., 2020). Arriving at a clear judgment would strengthen the influence of expected entrepreneurial benefits on academic entrepreneurial intention.

Second, with the security of the social network, academics are more optimistic about the potential returns from future entrepreneurial activities (Dai and Xu, 2021). Such positive thoughts would catalyze the academics who hold positive expectations of the entrepreneurial benefits to have a higher level of entrepreneurial intention. Accordingly, this paper proposes that:

*H5b*: Social network exerts a moderating effect on the relationship between expected entrepreneurial benefits and academic entrepreneurial intention.

**Figure 1:**
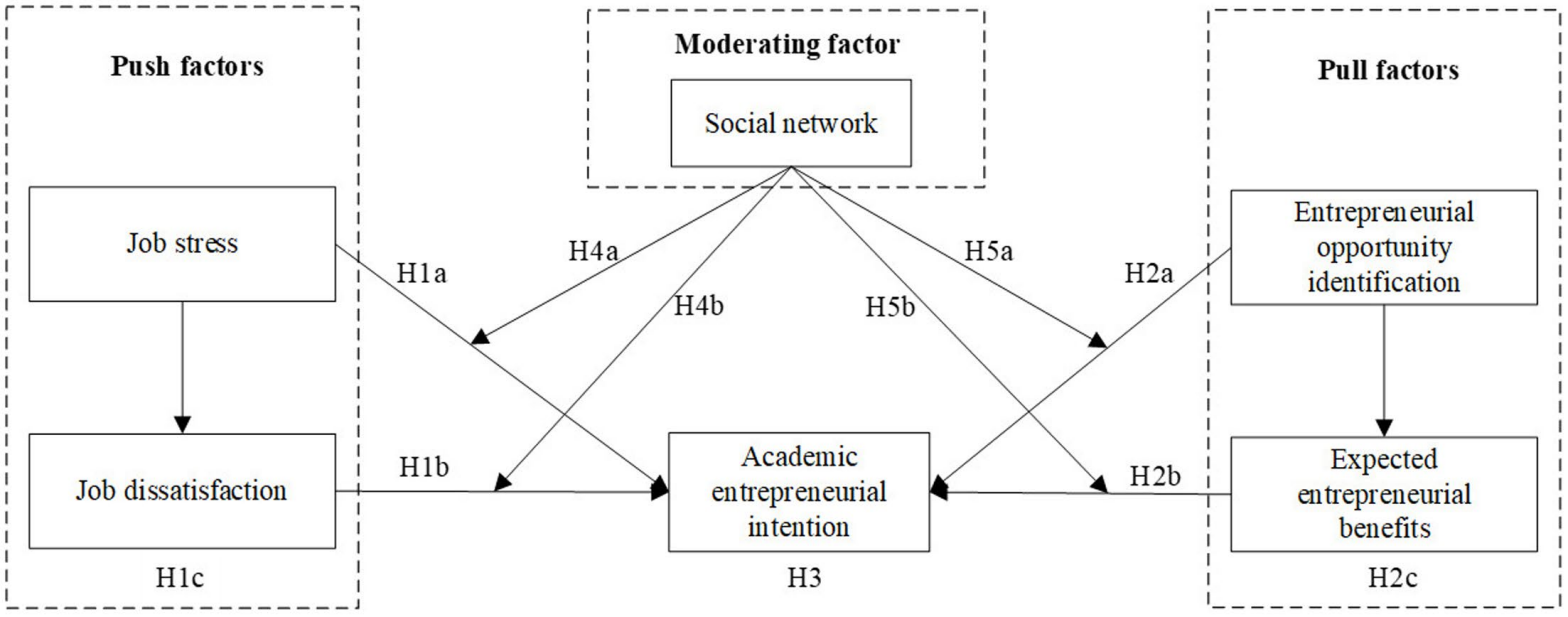
The theoretical framework.

The theoretical framework is shown in Figure 1.

## 3. Research methodology

### 3.1. Sample and data collection

Before data collection, ethical approval was obtained from the College Ethics Committee of Northeast Normal University. In addition, all participants were informed about the study purposes, voluntary participation, and the consent statement was obtained from all participants included in writing on the Likert Scale for the survey.

To evaluate the hypotheses developed above, data was collected from September 2020 to December 2020 in China. This study targeted Chinese academics for the following reasons. First, academic entrepreneurship has received substantial focus from Chinese government, and China has implemented a series of supportive policies to encourage academics to engage in entrepreneurial activities (Wu et al., 2022). Second, with an increasing number of universities in China adopting the “promotion or dismissal” system, academics, especially young academics, are under immense promotion pressure. Third, policy on academic entrepreneurship is poorly implemented, and the proportion of Chinese teachers engaging in academic entrepreneurship remained at a low level (Meng et al., 2018). Therefore, it is necessary to identify the factors affecting Chinese academics’ intentions to engage in entrepreneurship.

The questionnaire was collected through two channels, face-to-face questionnaire collection, and online questionnaire collection. In the beginning, the definition of academic entrepreneurship defined by Di Paola (2021) were described. By eliminating missing questionnaires and the questionnaires that were answered with a response time of fewer than 180 s, a total of 540 face-to-face questionnaires and 502 online questionnaires were confirmed finally. The answer time criteria were set based on the pilot study. The pilot results showed that to finish the entire questionnaire, 180 s was considered the minimum time. In addition, the test results of the ANOVA showed that no significant differences exist between the samples from the two channels.

The current study utilized the Harman single-factor test to examine the severity of the homology error. Results of the exploratory factor analysis indicated that the variance interpretation rate of the unrotated first principal component was 30.22%, which was less than 50%. This suggests that the common method deviation of the current research was not serious (Hair et al., 2010).

Specifically, Table 1 shows the distribution results of the 1,042 respondents.

**Table 1:** Distribution results of the samples (*n* = 1,042).

Demographic characteristics	Category	Percent (%)
Gender	Male	43	Female	57
Age	21–30	10	31–40	39	41–50	37	>50	14
Position	Professor	37	Associate professor	43	Lecturer	20
Research type	Basic research	33	Applied research	67
University level	Project 985 university	32	Project 211 university	35	Other	33
Academic discipline	Social science	32	Natural science	30	Engineering	29	Other	9

### 3.2. Measures

The measures of all constructs were 7-Likert scale and developed from the established literature with minor revisions to fit the context of the current research. In addition, these items are commonly adopted on the topic of academic entrepreneurship (Goethner et al., 2012; Foo et al., 2016; Neves and Brito, 2020).

*Job stress*. Following Raedeke and Smith (2004), Job stress was measured by the following items: (1) The current job is stressful, (2) The teaching workload is heavy, (3) The current research task is heavy, (4) Teacher evaluation and assessment make me feel a lot of pressure, (5) Work not related to my job takes up considerable time and energy.

#### 3.2.1. Job dissatisfaction

Based on Michigan Organizational Assessment Questionnaire proposed by Seashore et al. (1982), respondents were asked to indicate the extent to which they were dissatisfied with the following aspects of the job: (1) Work itself, (2) Salary, (3) Promotion, (4) Interpersonal relationship, (5) Work environment.

#### 3.2.2. Entrepreneurial opportunity identification

Reliance on the work of Kuckertz et al. (2017): Entrepreneurial opportunity identification was measured by the following items: (1) I am always alert to business opportunities, (2) I research potential markets to identify business opportunities, (3) I look for information about new ideas on products or services, (4) I regularly scan the environment for business opportunities.

#### 3.2.3. Expected entrepreneurial benefits

Adapting the proposal of Goethner et al. (2012), Expected entrepreneurial benefits was measured by the following items: (1) To increase personal income, (2) To promote social status, (3) To realize self-value, (4) To applicate and explore research outcomes, (5) To get a sense of achievement, (6) To gain personal freedom.

#### 3.2.4. Social network

Based on Goethner et al. (2012), Social network was measured by the following items: My contacts or discussions with (1) business friends; (2) people in government sector; (3) people in banks and financial institutions; (4) potential suppliers; (5) potential partners, could provide me with information and support that could help or encourage me to undertake a new venture.

#### 3.2.5. Academic entrepreneurial intention

Drawing on the scale developed by Linan and Chen (2009), Academic entrepreneurial intention was measured by the following items: (1) I am ready to do anything to be an entrepreneur, (2) My professional goal is to become an entrepreneur, (3) I will make every effort to start and run my own firm, (4) I am determined to create a firm in the future, (5) I have very seriously thought about starting a firm.

We controlled for six individual and work-related factors that could affect academic entrepreneurial intention, including gender, age, position, research type, university level, and academic discipline.

## 4. Empirical analysis and results

### 4.1. Reliability and validity test

The results of the reliability and validity test are shown in Table 2. The index of Cronbach’s α of all six constructs exceed 0.8, which indicates that the reliability of the scales used in the current research reaches the fundamental criteria.

**Table 2:** Results of reliability and validity test.

Construct	Items	Cronbach’s α	CR	AVE
Job stress	5	0.86	0.89	0.63
Job dissatisfaction	5	0.83	0.88	0.60
Entrepreneurial opportunity identification	4	0.90	0.93	0.77
Expected entrepreneurial benefits	6	0.88	0.91	0.64
Social network	5	0.91	0.93	0.60
Academic entrepreneurial Intention	5	0.94	0.95	0.81

CR, construct reliability; AVE, average variance extracted.

The scale used in the study has been widely adopted by existing literature, thus content validity is guaranteed. The values of average variance extracted (AVE) of all six constructs are greater than 0.5, which suggests that the convergence validity of the scales used in the current research reaches the fundamental criteria. In addition, the square root of AVE regarding all six constructs is greater than the correlation coefficients between each pair of constructs, which supports the notion that the discriminant validity of the scales used in the current research could be accepted (Hair et al., 2010).

### 4.2. Correlation analysis

The results of the correlation analysis are reported in Table 3.

**Table 3:** Results of correlation analysis.

Variable	1	2	3	4	5	6	7	8	9	10	11	12
1. Gender	**–**											
2. Age	0.08^*^	**–**										
3. Position	0.04	−0.54^**^	**–**									
4. Research type	−0.01	0.19^**^	−0.14^**^	**–**								
5. University level	0.14^**^	−0.15^**^	0.27^**^	0.02	**–**							
6. Academic discipline	−0.18^**^	0.01	0.07^*^	−0.09^**^	0.08^*^	**–**						
7. Job stress	−0.06	0.13^**^	0.03	0.14^**^	0.06	−0.02	**0.79**					
8. Job dissatisfaction	−0.12^**^	0.03	0.05	0.15^**^	0.14^**^	−0.01	0.66^**^	**0.77**				
9. Entrepreneurial opportunity identification	−0.21^**^	−0.07^*^	0.15^**^	0.16^**^	0.08^*^	0.11^**^	0.16^**^	0.28^**^	**0.88**			
10. Expected entrepreneurial benefits	−0.21^**^	−0.15^**^	0.14^**^	0.11^**^	0.15^**^	0.11^**^	0.24^**^	0.34^**^	0.60^**^	**0.8**		
11. Social network	−0.26^**^	−0.01	0.02	0.19^**^	0.01	−0.03	0.25^**^	0.29^**^	0.35^**^	0.48^**^	**0.77**	
12. Academic entrepreneurial Intention	−0.23^**^	−0.06^*^	0.18^**^	0.15^**^	0.17^**^	0.04	0.22^**^	0.35^**^	0.64^**^	0.65^**^	0.68^**^	**0.9**

The bold numbers on the diagonal are the square root of AVE. ^**^p < 0.01; ^*^p < 0.05.

As for control variables, academic entrepreneurial intention is positively correlated with position, research type, and university level, but negatively correlated with gender and age. As for the main variables, all five variables are significantly related to academic entrepreneurial intention, which initially verified the proposed hypothesis. Besides, there is no severe multi-collinearity among variables since none of the correlation coefficients exceeded 0.7.

### 4.3. Hypothesis test

#### 4.3.1. Direct effect test

In this paper, data analysis was conducted using SPSS 26.0 and MPLUS 7.0, whose applicability has been widely demonstrated in research (Zhang et al., 2019; Wang et al., 2020). Specifically, the direct effects of push and pull factors on academic entrepreneurial intention are examined with multiple regression analysis. The regression equations are constructed with academic entrepreneurial intention as the dependent variable, and the results of the regression analysis are shown in Table 4.

**Table 4:** The direct effects of push and pull factors.

Variable	Model 1	Model 2	Model 3	Model 4	Model 5	Model 6	Model 7
Gender	−0.193^***^	−0.164^***^	−0.139^***^	−0.116^***^	−0.099^***^	−0.139^***^	−0.081^***^
Age	0.026	0.041	0.060	−0.055	0.053	0.061	0.003
Position	0.232^***^	0.184^***^	0.190^***^	0.157^***^	0.196^***^	0.194^***^	0.165^***^
Research type	0.132^***^	0.112^***^	0.093^**^	0.023	0.053^**^	0.093^**^	0.015
University level	0.134^***^	0.123^***^	0.095^**^	0.117^***^	0.058^**^	0.093^**^	0.068^**^
Academic discipline	−0.011	0.004	0.013	−0.051^**^	−0.064^**^	0.013	−0.072^**^
Job stress		0.207^***^				−0.032	
Job dissatisfaction			0.291^***^			0.315^***^	
Entrepreneurial opportunity identification				0.560^***^			0.300^***^
Expected entrepreneurial benefits					0.625^***^		0.471^***^
Adjust *R*^2^	0.230	0.269	0.306	0.466	0.553	0.367	0.598
F-value	34.27^***^	37.88^***^	45.48^***^	91.73^***^	127.67^***^	70.23^***^	142.06^***^

^***^p < 0.01; ^**^p < 0.05.

The results indicated there is a significant positive relationship between job stress and academic entrepreneurial intention (β = 0.207, *p* < 0.01), H1a was supported. Job dissatisfaction exerts a significant positive effect on academic entrepreneurial intention (β = 0.291, *p* < 0.01), H1b is supported.

There is a significant positive relationship between entrepreneurial opportunity identification and academic entrepreneurial intention (β = 0.560, *p* < 0.01), H2a is supported. Expected entrepreneurial benefits exerts a positive effect on academic entrepreneurial intention (β = 0.625, *p* < 0.05), H2b is supported.

In addition, the current study applies the official stata statement “suest” to compare the significance of differences between path coefficient groups by pairing pull and push factors. The results show that the differences in the path coefficients are all significant and the effects of both pull factors are greater than those of push factors. Thus, H3 is supported.

#### 4.3.2. Mediating effect test

This paper firstly applies the stepwise regression analysis to test the mediating effects of job dissatisfaction and expected entrepreneurial benefits. The results are shown in Table 4.

In model 6, job dissatisfaction exerts a significant positive effect on academic entrepreneurial intention (β = 0.315, *p* < 0.01), and the effect of job stress are not significant (β = −0.032, *p* > 0.1). Thus, job dissatisfaction exerts a mediating effect between job stress and academic entrepreneurial intention, H1c is supported.

In model 7, expected entrepreneurial benefits exerts a significant positive effect on academic entrepreneurial intention (β = 0.471, *p* < 0.01). Moreover, compared to model 4, the coefficient of entrepreneurial opportunity identification on academic entrepreneurial intention declines from 0.560 to 0.300. This indicates that expected entrepreneurial benefits exert a mediating effect between entrepreneurial opportunity identification and academic entrepreneurial intention, H2c is supported.

Furthermore, Preacher and Hayes (2008) propose that there is a certain drawback in examining mediating effects with stepwise regression analysis, and the Bootstrap test can compensate for this shortcoming to some extent. Therefore, in this research, the Bootstrap approach is employed to revalidate the mediating effects of job dissatisfaction and expected entrepreneurial benefits. Basing on the view of Cheung and Lau (2008), a significant indirect effect would be confirmed if the 95% confidence interval excludes 0.

The results are shown in Table 5.

**Table 5:** Bootstrap test of mediating effect.

Path	Indirect effect	95% confidence interval	
		Lower	Upper
Job stress → Job dissatisfaction →Academic entrepreneurial intention	0.760 × 0.291 = 0.221	0.109	0.399
Entrepreneurial opportunity identification → Expected entrepreneurial benefits → Academic entrepreneurial intention	0.552 × 0.625 = 0.345	0.203	0.476

As for the mediating effect of job dissatisfaction, the 95% confidence interval excluding 0, indicating job dissatisfaction exerts a mediating effect between job stress and academic entrepreneurial intention, H1c is further supported.

As for the mediating effect of expected entrepreneurial benefit, the 95% confidence interval excluding 0, indicating expected entrepreneurial benefits play a mediating role in the relationship between entrepreneurial opportunity identification and academic entrepreneurial intention, H2c is further supported.

#### 4.3.3. Moderating effect test

This study utilizes stepwise regression analysis to test the moderating effect of social network. The results are shown in Table 6.

**Table 6:** The test of moderating effect.

Variable	Model 8	Model 9	Model 10	Model 11	Model 12	Model 13	Model 14	Model 15
Gender	−0.031	−0.042^**^	−0.019	−0.011	−0.004	−0.004	−0.016	−0.017
Age	0.114^**^	0.109^**^	0.123^***^	0.134^***^	0.040	0.043	0.107^**^	0.103^**^
Position	0.138^***^	0.150^***^	0.135^***^	0.112^***^	0.108^***^	0.111^***^	0.148^***^	0.141^***^
Research type	0.031	0.049^**^	0.021	0.039^*^	−0.030^*^	−0.028	0.008	0.004
University level	0.122^***^	0.116^***^	0.107^***^	0.097^***^	0.114^***^	0.119^***^	0.077^***^	0.072^***^
Academic discipline	0.038^*^	−0.005	0.043^**^	0.018	−0.001	0.004	−0.012	−0.025
Social network	0.650^***^	0.670^***^	0.629^***^	0.670^***^	0.565^***^	0.563^***^	0.490^***^	0.502^***^
Job stress	0.061^**^	0.060^**^						
Job dissatisfaction			0.140^***^	0.143^***^				
Entrepreneurial opportunity identification					0.408^***^	0.413^***^		
Expected entrepreneurial benefits							0.412^***^	0.411^***^
Social network * Job stress		0.118^***^						
Social network * Job dissatisfaction				0.140^***^				
Social network * Entrepreneurial opportunity identification						0.035		
Social network * Expected entrepreneurial benefits								0.051
Adjust *R*^2^	0.603	0.614	0.616	0.636	0.720	0.705	0.715	0.703
F-value	144.53^***^	151.75^***^	152.93^***^	163.05^***^	244.61^***^	226.12^***^	238.74^***^	223.59^***^

^***^p < 0.01; ^**^p < 0.05, ^*^p < 0.1.

The results of model 9 indicate that the interaction of job stress and social network positively affects academic entrepreneurial intention (β = 0.118, *p* < 0.01), and compare to model 8, both adjusted R^2^ and *F*-value increase significantly. Thus, social network plays a moderating role in the relationship between job stress and academic entrepreneurial intention, H4a is supported.

The results of model 11 indicate that the interaction of job dissatisfaction and social network positively affects academic entrepreneurial intention (β = 0.140, *p* < 0.01), and compare to model 10, both adjusted R^2^ and F-value increase significantly. Thus, social network plays a moderating role in the relationship between job dissatisfaction and academic entrepreneurial intention, H4b is supported.

The results of model 13 indicate that the effect of the interaction of entrepreneurial opportunity identification and social network on academic entrepreneurial intention is insignificant (β = 0.035, *p* > 0.1). Thus, social network do not play a moderating role in the relationship between entrepreneurial opportunity identification and academic entrepreneurial intention, H5a is not supported.

The results of model 15 indicate that the effect of the interaction of expected entrepreneurial benefits and social network on academic entrepreneurial intention is insignificant (β = 0.051, *p* > 0.1). Thus, social network do not play a moderating role in the relationship between expected entrepreneurial benefits and academic entrepreneurial intention, H5b is not supported.

**Figure 2:**
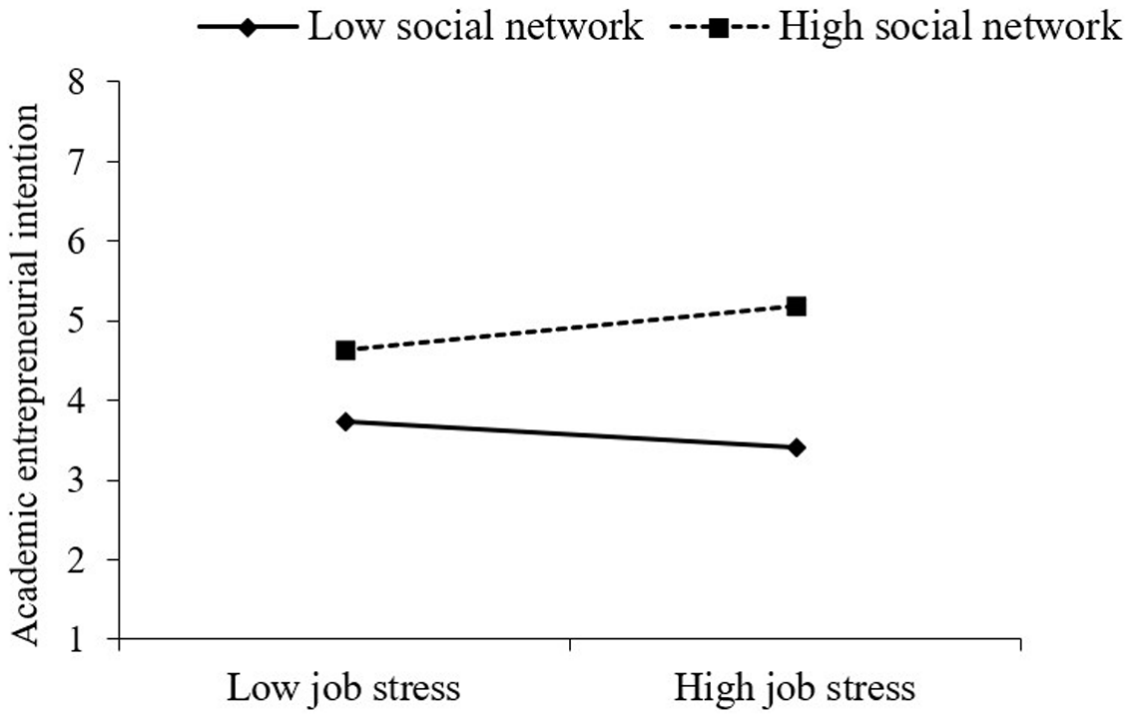
Moderating effects of social network on job stress influencing academic entrepreneurial intention.

**Figure 3:**
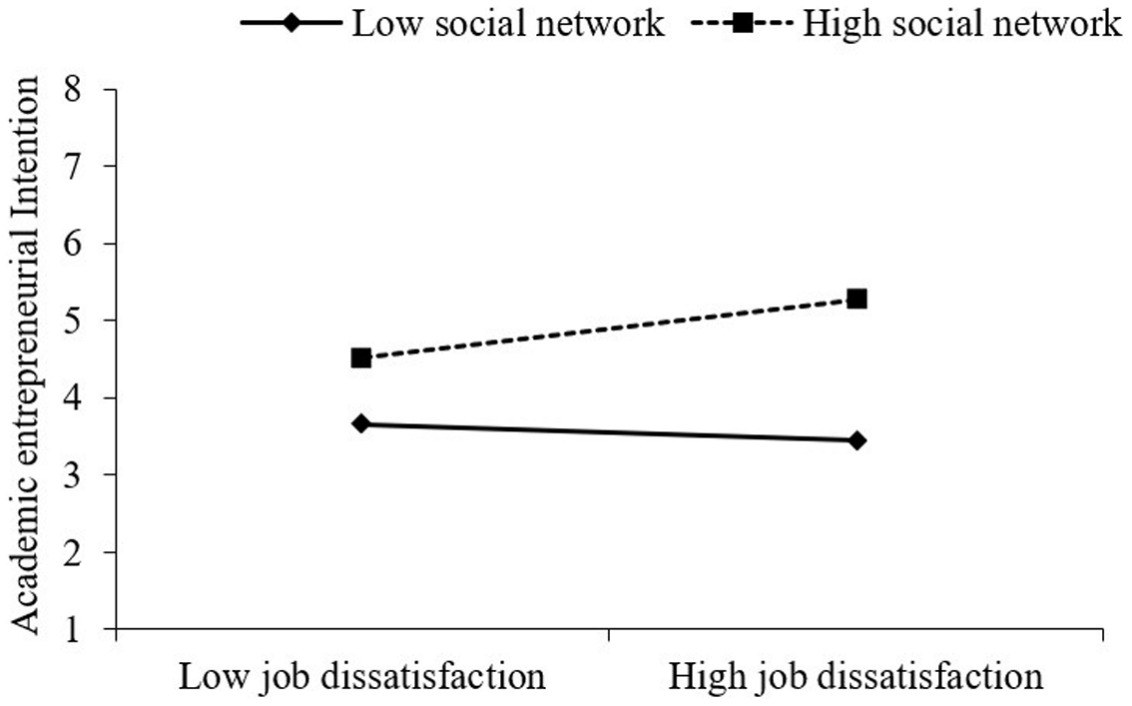
Moderating effects of social network on job dissatisfaction influencing academic entrepreneurial intention.

As shown in Figure 2, in the case of the high social network, the level of academic entrepreneurial intention is higher for a higher level of job stress. In Figure 3, in the case of the high social network, the slope of job dissatisfaction acting on academic entrepreneurial intention is steeper. Results indicate that social network exerts positive moderating effects on the relationship between job stress, job dissatisfaction, and academic entrepreneurial intention. H4a and H4b are further supported.

## 5. Discussion

The current study explores the formation process of academic entrepreneurial intention from the push-pull perspective, with job stress and job dissatisfaction as push factors, entrepreneurial opportunity identification, and expected entrepreneurial benefits as pull factors. This paper not only discusses the direct impact of push-pull factors on academic entrepreneurial intention but also examines the mediating role of job dissatisfaction and expected entrepreneurial benefits, clarifying the formation mechanism of academic entrepreneurial intention. In addition, the current research investigates the moderating role of social network, testing the boundary conditions it plays in the academic entrepreneurial intention formatting process as well. The key findings from the results are discussed below.

### 5.1. The effect of push factors

First, our results indicate that job stress positively influences academic entrepreneurial intention. The conclusion is consistent with the work of Obschonka et al. (2015), which revealed that employees may be “pushed” to engage in entrepreneurial activities when they experience high levels of job stress.

Second, our findings reveal that job dissatisfaction enhances academics’ entrepreneurial intentions. This conclusion is in line with the findings of Blaese et al. (2021), which concluded that academics may be motivated to engage in entrepreneurship if their current job is somewhat less satisfying than that which entrepreneurship might bring them.

Third, job dissatisfaction is identified as a mediator in the process by which job stress affects academic entrepreneurial intention. The relationship between job stress and job dissatisfaction has been confirmed in many fields (Bakker and Demerouti, 2017). For example, the work of Skaalvik and Skaalvik (2017) showed that job stress would reduce teachers’ satisfaction with their work which in turn results in a motivation to leave the teaching profession.

### 5.2. The effect of pull factors

First, our results reveal that entrepreneurial opportunity identification positively predicts academic entrepreneurial intention. The conclusion is consistent with the work of Aparicio et al. (2016), which revealed that employees may be “pulled” to engage in entrepreneurial activities when they identify valuable entrepreneurial opportunity.

Second, our research concludes that expected entrepreneurial benefits contribute to inspiring academic’s entrepreneurial intention. This could be attributed to the fact that academic entrepreneurial decision-making is a rational process of comparing payoffs and rewards (Thai et al., 2020). Besides, some researchers have come to similar conclusions which highlighted that expected entrepreneurial-related physical and spiritual benefits would attract academics to take part in entrepreneurship (Goethner et al., 2012; Neves and Brito, 2020).

Third, expected entrepreneurial benefits would also play a mediator in the relationship by which entrepreneurial opportunity identification affects academic entrepreneurial intention. This echoes the notion that “the nature of entrepreneurship is the process of identifying, evaluating and developing opportunities” (Wood et al., 2017). The identification of entrepreneurial opportunities and formulation of entrepreneurial ideas is the very first step toward the emergence of academic entrepreneurial intention (Clough et al., 2019). Then academics are able to proceed with the assessment of entrepreneurial returns for specific entrepreneurial opportunities, that is, the second step (Hahn, 2020). After identifying an entrepreneurial opportunity and realizing its potential benefits, academics then may embark on a desire to exploit it and engage in entrepreneurial activities (Vandor and Franke, 2016).

### 5.3. The comparison of push and pull effects

Interestingly, we also get some intriguing findings regarding the comparison of push and pull effects. The test results show that pull factors play a more powerful role than push factors.

There are several reasons to explain this notion. First, starting a new business without identifying rewarding opportunities, simply for escaping the negative influences of the current job, would expose academics to a high level of venture risk (Urban and Chantson, 2019). In such cases, the propensity of academics choosing to engage in academic entrepreneurial activity is likely to be thwarted and discouraged by concerns about the high-level risk. Second, entrepreneurial activity needs to be undertaken with a specific entrepreneurial idea (Aparicio et al., 2016). Academics without identifying a suitable entrepreneurial opportunity, or perceiving entrepreneurial benefits are attractive enough, solely driven by negative job-related factors, may not choose to engage in entrepreneurship. Instead, they may select to pursue other careers. Under such circumstances, academics’ intention to pursue academic entrepreneurship is likely to remain low. This founding provides a theoretical reference for the government to design more targeted policies to motivate academics to engage in entrepreneurship.

### 5.4. The moderating effect of social network

Our findings further confirm the positive moderating role of social network in the relationships between job stress and job dissatisfaction on academic entrepreneurial intention. This could be explained by the fact that entrepreneurship among academics is different from entrepreneurship by general employees because academics own some research achievements that can be translated and commercialized (Wu et al., 2015; Diánez-González and Camelo-Ordaz, 2019; Urban and Chantson, 2019). Thus, in the case of academics pushed by negative job-related factors to consider engaging in entrepreneurship, the availability of the key entrepreneurial resources and valuable information from social network would enhance their ambition to enter entrepreneurship (Dai and Xu, 2021).

However, the moderating effect of the social network in the influencing process between entrepreneurship-related pull factors and academic entrepreneurial intention is not validated. This is because, the identification of entrepreneurial opportunity reflects sharp entrepreneurial alertness and unusual information sifting and filtering abilities, which is crucial to entrepreneurial success (Dai and Xu, 2021). Academics with these abilities will manage to achieve their entrepreneurial aims regardless of the lack of resources (Wood et al., 2017). On the other hand, academics may be blinded by the expected lucrative benefits of entrepreneurship and develop a high-level ambition, regardless of objective resource constraints. Since there is no cost to the emergence of intentions, no need to calculate feasibility carefully (Gieure et al., 2020). Thus, social network may have a stronger catalytic or restrictive role in the transformation from intention to behavior, rather than the academic entrepreneurial intention.

## 6. Implication

### 6.1. Theoretical implication

The theoretical implications of the current research are as follows. First, this study advances the academic entrepreneurship literature by proposing a comprehensive framework under a dual “push-pull” perspective and comparing the direct effect of push-pull factors on academic entrepreneurial intention. Besides, this research provides a deeper and broader insight into the academic entrepreneurship literature by and exploring the influencing mechanism in the process. Existing research regarding academic entrepreneurial intention has predominantly focused on attractive entrepreneurship-related explanations. Few studies have focused on the significant role of push factors regarding academics’ current jobs in the formation process of academic entrepreneurial intention. Dressing on this gap, this study explores the impact of push factors including job stress and job dissatisfaction, and pull factors including entrepreneurial opportunity identification and expected entrepreneurial benefits on academic entrepreneurial intention, which provides a more complete picture of academic entrepreneurial intention.

Second, we not only incorporate push-pull factors into a research framework but also compared their impacts. Results indicate that pull factors are the primary force in igniting academic entrepreneurial intention, while push factors play a subordinate role. This is consistent with the results of Elfenbein et al. (2010), which integrated the push-pull factors affecting employee entrepreneurship and found that scientists’ venture decisions were dominated by pull factors. Furthermore, we have responded to the call by Neves and Brito (2020) to enrich and reassert the powerful foundation role of pull factors in stimulating scholars’ willingness to engage in entrepreneurship. In addition, we clarify the mechanisms of push-pull factors influencing academic entrepreneurial intention by exploring the mediating effects of job dissatisfaction and expected entrepreneurial benefits.

Third, the current study aspires to offer contributions to social network theory. Existing research has mainly guided by social network theory to explore the direct impact of social network on academic entrepreneurial intention (Balven et al., 2018; Dai and Xu, 2021). However, few studies have focused on the moderating role of social network in the emergence of academic entrepreneurial intention, leaving an incomplete understanding of the role of social network within the academic entrepreneurial process. Through discussing “How do social network act in the formation process of academic entrepreneurial intention,” we reveal the differences regarding the moderating effects of social network on push-pull factors affecting academic entrepreneurial intention. As a result, we may provide a broader perspective and shed light on a new look at the role that social network theory plays in academic entrepreneurship.

### 6.2. Practical implication

The findings of the current study also offer some practical implications. First, universities should pay attention to and fully utilize the role of Technology Transfer Offices, organizing entrepreneurship-related training and lectures to expand the social network of scholars (Toniolo et al., 2020). Since, according to our research, the rich social network is instrumental in catalyzing the emergence of academic entrepreneurial intention. With the Technology Transfer Office as a bridge, universities offer academics access to investors and financing channels to enhance their intentions to engage in academic entrepreneurial activities (Balven et al., 2018; Yi, 2021).

Second, scholars in universities should emphasize on nurturing a sense of commercializing their research achievements (Li et al., 2020; Perkmann et al., 2021). To make maximum utilization of their research achievements, instead of treating papers and project applications as the end of the research. Academics should also maintain a high grade of entrepreneurial alertness, and be proactive in identifying the potential entrepreneurial opportunities inherent in their research. In addition, academics should actively build and keep in touch with enterprises through the process of collaborating with them, to gain access to entrepreneurship-related resources and information (Rippa and Secundo, 2018). Besides, academics shall promptly follow and participate in industry-academia-research cooperation conferences in their research areas and communicate sufficiently with investors and entrepreneurs to understand the application prospects of their research outcomes.

## 7. Limitation and future research

Our study has some limitations that should be noted. First, this study is conducted by Chinese scholars, and the generalizability of the findings may be limited to some degree. Considering the high degree of the institutional and cultural uniqueness of Chinese universities compared to universities from other countries, future research could be examined cross-nationally to further test the results of this study. Second, the current research mainly explores the influential factors and formation mechanisms of academic entrepreneurial intention, without focusing on the transformation process from intention to behavior. Nevertheless, there is still an enormous range of uncertainties from the emergence of entrepreneurial intention to the generation of entrepreneurial behavior (Yi, 2021). In the future, additional efforts should be devoted to exploring the key factors that influence the shifting process of academic entrepreneurial intention into academic entrepreneurial behavior, completing the picture of academic entrepreneurship research. Third, this study used a cross-sectional questionnaire design, collecting data in a single-time point, which could lead to questionable causality inference results. In order to address this issue, future research may consider longitudinal studies to collect data in different timepoint and verify the causal relationships proposed in this paper more precisely.

## 8. Conclusion

First, as for push effects, our results reveal that job stress positively predicts academic entrepreneurial intention, which indicates that academics in stressful work environments are more likely to seek ways to escape or defend themselves from such negative external stimuli and to engage in academic entrepreneurial activities on their own merits. In addition, job dissatisfaction enhances academics’ intentions to engage in entrepreneurial activities. This may be caused by the fact that dissatisfaction with the career-related or income-related elements has led academics to re-examine and plan their career paths. Further, job dissatisfaction is identified as a mediator in the process by which job stress affects academic entrepreneurial intention.

Second, as for pull effects, our results reveal that entrepreneurial opportunity identification positively predicts academic entrepreneurial intention, which indicates that academics identified valuable opportunities are more likely to seek ways to engage in academic entrepreneurial activities. Besides, expected entrepreneurial benefits contribute to inspiring academic’s entrepreneurial intention. After weighing various aspects, if academics believe that the rewards of participating in entrepreneurship outweigh the payoffs, as rational decision-makers, they would be more inclined to engage in academic entrepreneurial activities. In addition, expected entrepreneurial benefits also play a mediator in the relationship between entrepreneurial opportunity identification and academic entrepreneurial intention.

Third, our findings also point to some intriguing findings regarding the comparison of push and pull effects. The test results show that both push and pull factors do contribute to boosting academic entrepreneurial intention. Particularly, pull factors play a more powerful role than push factors.

Finally, as for the moderating role of social network, our findings confirm the positive moderating role of social network in the relationships between job stress and job dissatisfaction on academic entrepreneurial intention. This offers a fresh perspective to existing research which emphasizes the direct role of social network on academic entrepreneurial intention in the majority of cases. However, the moderating effect of the social network in the influencing process between identified entrepreneurial opportunity and expected entrepreneurial benefits on academic entrepreneurial intention are not validated. This conclusion enlightens universities and academics should fully appreciate and exploit the role of social network in stimulating academics’ entrepreneurial ambitions.

## Data availability statement

The raw data supporting the conclusions of this article will be made available by the authors, without undue reservation.

## Author contributions

ZY and KL designed the questionnaires, collected the data, and revised the manuscript. ZY analyzed the results and wrote the manuscript. All authors designed the study, decided to approve the final work, and took full responsibility for the originality of the research.

## Conflict of interest

The authors declare that the research was conducted in the absence of any commercial or financial relationships that could be construed as a potential conflict of interest.

## Publisher’s note

All claims expressed in this article are solely those of the authors and do not necessarily represent those of their affiliated organizations, or those of the publisher, the editors and the reviewers. Any product that may be evaluated in this article, or claim that may be made by its manufacturer, is not guaranteed or endorsed by the publisher.
